# Spectral CT quantitative parameters for predicting complete ablation after radiofrequency ablation in liver tumors: a retrospective study

**DOI:** 10.3389/fonc.2026.1797989

**Published:** 2026-06-01

**Authors:** Bin Zhang, Xiaoli Ge

**Affiliations:** 1 Department of Medical Imaging, Jinhua Central Hospital, Jinhua, Zhejiang, China; 2 Department of Ultrasound Diagnosis and Treatment, Jinhua Central Hospital, Jinhua, Zhejiang, China

**Keywords:** complete ablation, liver tumors, quantitative parameters, radiofrequency ablation, retrospective study, spectral CT

## Abstract

**Background:**

Radiofrequency ablation (RFA) is a key minimally invasive treatment for liver tumors, but predicting complete ablation remains challenging.

**Objective:**

This study aimed to evaluate the correlation between spectral CT quantitative parameters and complete ablation after RFA for liver tumors.

**Methods:**

This retrospective study enrolled 109 patients who underwent RFA for liver tumors and completed preoperative spectral CT scans (January 2022-October 2025). Patients were divided into complete ablation (n=81) and incomplete ablation (n=28) groups based on postoperative MRI. Preoperative spectral CT parameters [normalized iodine concentration (NIC), spectral curve slope (λ-HU), effective atomic number (Z-eff)] in arterial and portal venous phases were measured. Clinical factors including tumor size, AFP, and vessel adjacency were collected. Logistic regression and ROC analysis were used to build a predictive model.

**Results:**

The arterial phase NIC, λ-HU, and Z-eff in the lesion parenchymal area of the complete ablation group were significantly lower than those in the incomplete ablation group (P<0.05). Additionally, the proportions of patients with preoperative serum alpha-fetoprotein (AFP) >100 ng/mL, tumors adjacent to major intrahepatic vessels, and a maximum tumor diameter >3 cm were significantly lower in the complete ablation group compared to the incomplete ablation group (P<0.05). Logistic regression analysis identified maximum tumor diameter >3 cm (OR = 8.50, 95% CI: 1.38-52.50), preoperative AFP >100 ng/mL (OR = 9.69, 95% CI: 1.34-70.15), tumor adjacency to major intrahepatic vessels (OR = 12.90, 95% CI: 1.34-124.21), arterial phase NIC (OR = 5.66, 95% CI: 1.49-21.57), λ-HU (OR = 4.32, 95% CI: 1.50-12.47), and Z-eff (OR = 5.12, 95% CI: 1.18-22.22) as independent predictive factors. The combined predictive model constructed from these factors achieved an AUC of 0.933 (95% CI: 0.869-0.972) for predicting complete ablation, with a predictive performance superior to that of any single parameter (P<0.001).

**Conclusion:**

Spectral CT quantitative parameters, along with tumor size, vessel adjacency, and AFP level, are significantly associated with complete ablation after RFA for liver tumors and can serve as predictive biomarkers for optimizing individualized treatment.

## 1 Introduction

Primary liver cancer, which originates from hepatocytes or intrahepatic bile duct epithelial cells, is one of the most common and highly malignant tumors worldwide (1). Furthermore, the liver is the most frequent site of metastasis for gastrointestinal malignancies such as colorectal cancer (2, 3). For patients with early-stage liver tumors, surgical resection was once considered the primary curative treatment option (4). However, the majority of patients are diagnosed at intermediate or advanced stages in clinical practice, or they may have underlying conditions such as cirrhosis, hepatic or renal insufficiency, or coagulation disorders that preclude tolerance for traditional open surgery or laparoscopic resection. Consequently, minimally invasive treatment techniques have gradually emerged as a core therapeutic choice for these patients (5).

Radiofrequency ablation (RFA) is a well-established minimally invasive interventional therapy. It is also widely used for palliative treatment of intermediate to advanced liver tumors to control disease progression and prolong patient survival (6–8). Compared to traditional surgery, RFA effectively preserves the function of normal liver parenchyma and reduces the risk of postoperative liver failure. This makes it particularly suitable for patients with liver tumors in the setting of underlying cirrhosis (9). Although RFA is widely used in the treatment of liver tumors, ablation completeness remains a critical factor influencing therapeutic efficacy and patient prognosis (10). Incomplete ablation signifies residual tumor tissue or an insufficient ablation margin. This condition readily leads to local tumor recurrence and distant metastasis, significantly reducing patients’ disease-free survival and overall survival (11). Therefore, accurately evaluating ablation completeness and promptly identifying the risk of incomplete ablation are of paramount importance for optimizing preoperative treatment planning, adjusting intraoperative strategies, and formulating postoperative follow-up protocols.

In terms of efficacy evaluation, accurately assessing complete ablation remains a clinical challenge. Postoperative contrast-enhanced CT or MRI, the gold standard for treatment response, relies on the absence of arterial-phase enhancement within the ablation zone. However, early after RFA, an inflammatory reaction band at the ablation margin appears as rim-like enhancement, which is difficult to distinguish from residual tumor or early recurrence. This often delays the optimal window for efficacy assessment and timely supplemental treatment (12, 13). Therefore, using non-invasive imaging before RFA to identify factors influencing ablation outcomes and predict the likelihood of complete ablation would be clinically valuable. Such an approach could help optimize individualized treatment plans, select the most appropriate therapeutic strategy, and guide decisions on combining other modalities to improve success rates.

In recent years, spectral CT imaging technology, as an emerging functional imaging tool, has offered a novel perspective for addressing the aforementioned clinical challenges (14). Unlike conventional CT, which provides only morphological information based on X-ray attenuation, spectral CT acquires data at two different energy levels. By leveraging the principle that materials exhibit different attenuation characteristics under X-rays of varying energies, it enables material decomposition and quantitative analysis (15). This technology can generate a series of quantitative parameter maps, such as iodine density (ID) maps, effective atomic number (Z-eff) maps, and spectral curve slope (λ-HU) maps, among others (16). These quantitative parameters go beyond the simple “grayscale” concept of traditional imaging, offering the potential for a deeper understanding of the intrinsic biological behavior of tumors from functional and material composition perspectives.

To address current clinical and research needs, this work aims to retrospectively correlate preoperative spectral CT findings with clinical data in patients who received RFA for liver tumors. The analysis will focus on investigating the correlation between preoperative spectral CT quantitative parameters and the postoperative imaging-confirmed status of complete ablation. Concurrently, incorporating clinical risk factors such as tumor size and location, this study seeks to construct and validate a multi-parameter integrated prediction model. The ultimate objective is to provide a theoretical foundation and clinical reference for pre-therapeutic assessment of the likelihood of achieving complete RFA ablation, identification of optimal candidates, early warning for high-risk cases, and optimization of individualized surgical pathway planning and treatment decision-making in clinical practice.

## 2 Methods

### 2.1 Study subjects

**Figure 1:**
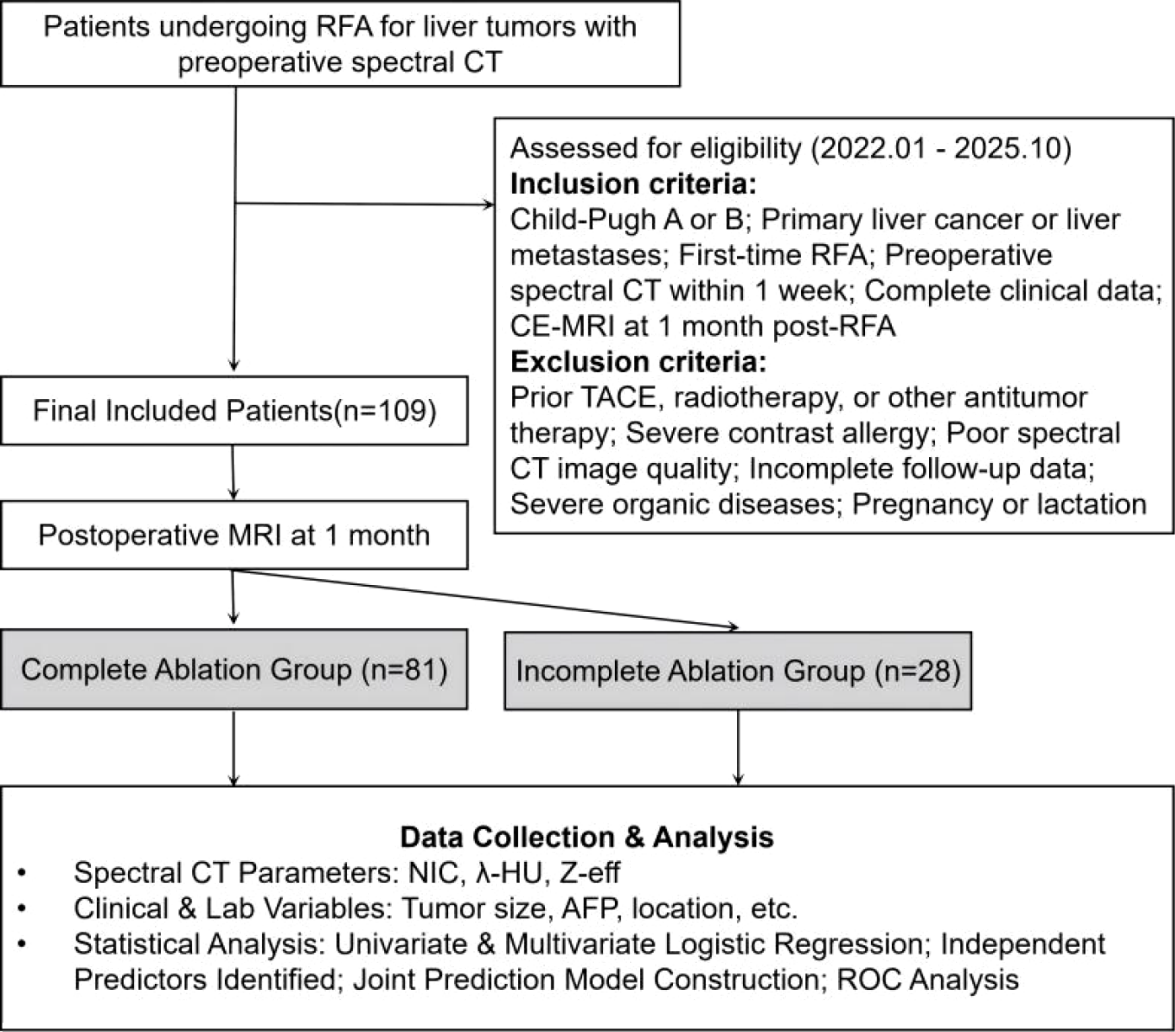
Research process.

This single-center, retrospective study analyzed patients undergoing hepatic tumor RFA between January 2022 and October 2025. Based on the results of regular postoperative imaging follow-up, patients were categorized into a complete ablation group and an incomplete ablation group, using the standardized terminology for image-guided tumor ablation (17). The study protocol was approved by the Ethics Committee of our hospital. The detailed study flowchart is presented in **Figure 1**.

### 2.2 Criteria

Inclusion criteria: (1) Child-Pugh class A or B, or patients whose liver function was optimized to this level through standard-of-care management before ablation; (2) Diagnosis of primary liver cancer or liver metastases; (3) Undergoing RFA treatment for the first time; (4) Completion of a standardized contrast-enhanced spectral CT scan within one week before the procedure, with image quality meeting the requirements for post-processing analysis; (5) Availability of complete baseline clinical data, including age, gender, liver function, tumor markers, etc.; (6) Performance of CE-MRI one month after RFA as the gold standard for efficacy evaluation.
Exclusion criteria: (1) Previous history of preoperative treatments such as transarterial chemoembolization (TACE), radiotherapy, or other anti-tumor therapies; (2) History of severe allergic reaction to CT contrast agents; (3) Presence of severe motion artifacts or noise in the spectral CT images, precluding accurate measurement of quantitative parameters; (4) Incomplete clinical or imaging follow-up data, making it impossible to definitively determine ablation efficacy; (5) Poor general condition of the patient, or presence of severe organic diseases affecting the heart, brain, lungs, kidneys, or other major organs, constituting surgical contraindications; (6) Pregnancy or lactation.

### 2.3 Treatment methods

Ablations were conducted using the Covidien 11c CTRF 220 RFA system. Patients were placed in the supine position in the interventional catheterization suite. Following local anesthesia, conventional ultrasound examination was performed. The puncture site, needle trajectory, and depth were determined through discussion. Under the guidance of a Philips EPIQ 7C color Doppler ultrasound system, a bipolar electrode RFA needle was used for puncture.

When the tip of the RFA needle reached the margin of the lesion, the RFA electrodes were sequentially activated based on the lesion size, and different radiofrequency programs and power settings were selected. After the needle tip reached the target area within the lesion, the needle was retracted approximately 0.5 cm. The ablation tines were then deployed according to the lesion dimensions to achieve an ablation zone encompassing the lesion and at least a 1 cm margin of surrounding normal tissue. This margin target was applied uniformly to all tumors, including both primary liver cancer and liver metastases, as standardization of the ablation protocol was prioritized in this retrospective study. The radiofrequency generator was activated, with the ablation time typically set for about 10 minutes. Upon completion of the ablation, tract ablation was performed to prevent seeding metastasis of cancer cells along the needle path.

Throughout the procedure, real-time monitoring was conducted using the Philips EPIQ 7C color Doppler ultrasound to observe the ablation effect. If residual tumor tissue was detected, additional ablation was performed to ensure optimal therapeutic outcome. Hemostatic, sedative, and analgesic medications were administered intravenously as necessary.

After the procedure, patients returned to the ward and were kept on strict bed rest. Routine postoperative care was provided, including cardiac monitoring, oxygen therapy, hepatoprotective agents, acid suppression, and fluid replacement. Liver function tests, blood biochemistry, and complete blood counts were rechecked. Patients were closely monitored for vital signs. Potential complications following RFA, such as bleeding at the puncture site, pain, fever, nausea, vomiting, and abdominal distension, were monitored for early detection and symptomatic management to prevent rare but life-threatening complications.

### 2.4 Imaging protocol and image analysis

1. Preoperative Spectral CT Scanning: Abdominal non-contrast, arterial phase, and portal venous phase contrast-enhanced scans were performed using a GE Revolution 256-slice spiral CT scanner in spectral imaging mode. Prior to the examination, all patients underwent bowel preparation. On the day of the scan, the procedure and precautions were reconfirmed with the patient. Metal objects were removed to minimize artifacts, and patients were instructed in breath-holding techniques. The patient was positioned supine, feet first. The scanning range extended from the diaphragmatic dome to the level of the lower poles of both kidneys. Scanning parameters included rapid tube voltage switching between 80 kVp and 140 kVp, automatic tube current modulation, a rotation speed of 0.8 seconds per rotation, a slice thickness of 5 mm, and an interval of 5 mm. For the contrast-enhanced phases, a dual-syringe power injector was used to administer the non-ionic iodinated contrast agent Iohexol via the right antecubital vein at a flow rate of 3-3.5 mL/s, with a total dose of 60–80 mL. Arterial phase scanning was triggered using the Smart Prep technique by dynamically monitoring the enhancement in the abdominal aorta. Monitoring began 10 seconds after the start of contrast injection, repeated every 2 seconds, and automatically triggered when the attenuation in the abdominal aorta reached 100 Hounsfield Units (HU). Portal venous phase scanning commenced 25–30 seconds after the arterial phase. The scanning range covered the entire upper abdomen, including the liver and both kidneys.
2. Data Processing: The acquired data were transferred to a backend NVIDIA RTX A6000 GPU workstation for analysis using GSI Viewer software. Regions of interest (ROIs) were selected by a radiologist with over five years of experience in diagnostic imaging. ROIs were placed within the solid component of the lesion and replicated in the immediate adjacent superior and inferior slices. Measurements were performed three times and averaged. ROIs were positioned to encompass as much solid tumor tissue as possible while avoiding cystic, necrotic, or hemorrhagic areas. The ROI area was kept consistent across the three selected slices. When measuring the surrounding liver parenchyma, care was taken to avoid large intrahepatic vessels and areas with prominent beam-hardening artifacts. The following parameters were obtained, measured, or calculated: ID: ID-lesion (mg/cm³) measured within the solid tumor component, and ID-aorta (mg/cm³) measured in the abdominal aorta at the same level. NIC=ID-lesion/ID-aorta. λ-HU=(CT40 keV - CT90 keV)/(90 - 40), where CT40 keV and CT90 keV represent the monochromatic CT values (in HU) of the lesion ROI at 40 keV and 90 keV, respectively. Z-eff: The Z-eff value recorded from the solid tumor component ROI.

To assess the reproducibility of spectral CT quantitative parameters, twenty patients were randomly selected from the study cohort. For intra-observer reproducibility, the same radiologist repeated the ROI measurements on the same spectral CT images after a two-week interval. For inter-observer reproducibility, a second independent radiologist (with five years of experience in abdominal imaging), blinded to the original measurements and clinical outcomes, performed ROI placement following the same protocol. The intraclass correlation coefficient (ICC) was calculated for NIC, λ-HU, and Z-eff in the arterial phase. The results showed excellent reproducibility for all parameters: intra-observer ICCs ranged from 0.92 to 0.95, and inter-observer ICCs ranged from 0.91 to 0.94 (all P<0.001). These findings confirm that the quantitative spectral CT measurements used in this study are highly reproducible.

### 2.5 Follow-up and efficacy evaluation criteria

Follow-up Protocol: All patients underwent contrast-enhanced MRI (GE 3.0T Signa Pioneer) examination one month after the RFA procedure as the primary time point for efficacy assessment.

Efficacy Evaluation was based on the revised standardized terminology reported by Ahmed et al. (17). Technical Effectiveness (Complete Ablation): On contrast-enhanced imaging, the original tumor area showed no nodular, mass-like, or irregular enhancement within or at the margin of the ablation zone, indicating complete necrosis of the tumor tissue. This finding was classified as “complete ablation” (i.e., technically effective treatment). Incomplete Ablation (Residual/Recurrent Tumor): The presence of nodular, patchy, or other forms of abnormal enhancement within or at the margin of the ablation zone on contrast-enhanced MRI, suggesting residual unablated tumor or early local tumor progression. This finding was classified as “incomplete ablation” (i.e., technically ineffective treatment). Based on the imaging findings at one month postoperatively, patients were categorized into a Complete Ablation group (technically effective) and an Incomplete Ablation group (technically ineffective).

### 2.6 Sample size estimation

Sample size estimation was performed using G*Power 3.1 software. Based on preliminary experiments and a review of relevant literature, the key quantitative parameter “Arterial phase NIC” was selected as the primary outcome measure. We set a two-tailed α=0.05 and a statistical power (1-β)=0.80. According to data from previous studies (18), we anticipated a significant difference in NIC between the Complete and Incomplete Ablation groups (effect size d ≥ 0.5), with an estimated sample ratio of approximately 3:1 between the groups. Calculation based on the independent samples t-test indicated that to achieve the preset statistical power, a minimum of 20 patients in the Incomplete Ablation group and 60 patients in the Complete Ablation group were required, totaling at least 80 patients. This study ultimately enrolled 109 patients (28 in the Incomplete Ablation group and 81 in the Complete Ablation group), exceeding the minimum sample size requirement and ensuring sufficient statistical power for the study results.

### 2.7 Statistical analysis

Data analysis was performed using SPSS 26.0 and R 4.2.1 software. Continuous variables, reported as mean ± SD or median (IQR), were compared using the t−test or Mann−Whitney U test based on distribution. Categorical variables, expressed as n (%), were compared with the Chi−square or Fisher’s exact test. Significant univariate predictors (P<0.05) were entered into a multivariate logistic regression to identify independent risk factors for incomplete ablation, from which a combined model was derived. To reduce overfitting bias, internal validation was performed using 10-fold cross-validation (repeated 5 times) and bootstrap resampling (200 iterations) to derive optimism-corrected performance estimates. Formal calibration metrics (e.g., Hosmer-Lemeshow test, calibration plot) were not computed given the limited number of incomplete ablation events (n=28), as such analyses would be statistically underpowered and potentially misleading. A nomogram was constructed based on the final multivariate model to provide a clinically usable tool for predicting incomplete ablation risk, with a corresponding point-based scoring system. Predictive performance was evaluated via ROC curves, calculating AUC, sensitivity, and specificity, along with cross-validated AUC. P<0.05 indicated statistical significance.

## 3 Results

### 3.1 Clinical characteristics

**Table 1** showed that there were no statistically significant differences between the groups regarding gender, age, Child-Pugh liver function classification, tumor location, history of cirrhosis, number of tumors, differentiation grade, etiology of liver disease, or pathological type (all P>0.05). However, the proportion of patients with a maximum tumor diameter>3 cm and the proportion of patients whose tumors were adjacent to major intrahepatic vessels were significantly higher in the Incomplete Ablation group compared to the Complete Ablation group (P<0.05).

**Table 1:** Comparison of clinical data.

Variables		Complete ablation group(n=81)	Incomplete ablation group(n=28)	Statistic	*P*
Gender (n, %)	male	56 (69.14)	22 (78.57)	χ²=0.910	0.340
	female	25 (30.86)	6 (21.43)		
Age ( $\bar{x}\pm s$, years)		53.44 ± 10.53	55.71 ± 8.59	t=-1.027	0.307
BMI ( $\bar{x}\pm s$, kg/m^2^)		22.46 ± 2.15	22.87 ± 1.78	t=0.905	0.367
Child-Pugh (n, %)	A	61 (75.31)	21 (75.00)	χ²=0.001	0.974
	B	20 (24.69)	7 (25.00)		
Location (n, %)	Left lobe of liver	39 (48.15)	13 (46.43)	χ²=0.025	0.875
	Right lobe of liver	42 (51.85)	15 (53.57)		
History of cirrhosis (n, %)		49 (60.49)	20 (71.43)	χ²=1.071	0.301
Tumor size ( $\bar{x}\pm s$, cm)	≤3	67 (82.72)	12 (42.86)	χ²=16.572	0.001
	>3	14 (17.28)	16 (57.14)		
Tumor number (n, %)	≤2	43 (53.09)	13 (46.43)	χ²=0.369	0.543
	>2	38 (46.91)	15 (53.57)		
Degree of differentiation (n, %)	I+II	40 (49.38)	12 (42.86)	χ²=0.355	0.551
	III+IV	41 (50.62)	16 (57.14)		
Cause of liver disease (n, %)	Hepatitis B	55 (67.90)	18 (64.29)	χ²=0.131	0.937
	Hepatitis C	16 (19.75)	6 (21.43)		
	Others	10 (12.35)	4 (14.29)		
Pathological types (n, %)	Hepatocellular carcinoma	61 (75.31)	21 (75.00)	–	0.926
	Intrahepatic cholangiocarcinoma	10 (12.35)	3 (10.71)		
	Colorectal origin	8 (9.88)	4 (14.29)		
	Neuroendocrine origin	2 (2.47)	0 (0.00)		
Adjacent to major intrahepatic vessels (n, %)		20 (24.69)	15 (53.57)	χ²=7.961	0.005

t, t-test; χ², Chi-square test; -, Fisher exact; BMI, Body Mass Index.

### 3.2 Serological indicators

A comparison of blood biochemical and tumor marker-related indicators between the two patient groups is presented in detail in **Table 2**. There was a significant difference in the distribution of serum alpha-fetoprotein (AFP) levels between the two groups. The proportion of patients with an AFP level>100 ng/mL was significantly higher in the Incomplete Ablation group compared to the Complete Ablation group (P = 0.007).

**Table 2:** Comparison of serological indicators.

Variables	Complete ablation group(n=81)	Incomplete ablation group(n=28)	Statistic	*P*
White Blood Cells (×10^9^/L)	5.37 ± 2.09	5.48 ± 1.98	t=-0.222	0.825
Neutrophils (×10^9^/L)	2.59 ± 1.27	2.65 ± 1.35	t=-0.247	0.806
Lymphocytes (×10^9^/L)	1.45 ± 0.65	1.39 ± 0.73	t=0.407	0.685
Monocytes (×10^9^/L)	0.37 ± 0.17	0.41 ± 0.19	t=-0.936	0.351
Platelets (×10^9^/L)	103.11 ± 33.13	98.45 ± 32.11	t=0.647	0.519
Albumin (g/L)	36.45 ± 5.33	35.42 ± 6.71	t=0.816	0.417
Total Bilirubin (μmol/L)	17.16 ± 4.69	18.66 ± 5.45	t=-1.390	0.167
Alpha-fetoprotein (AFP) (ng/mL)				
≤100	47 (58.02)	8 (28.57)	χ²=7.221	0.007
>100	34 (41.98)	20 (71.43)		
Carcinoembryonic Antigen (ng/mL)	6.10 (4.60, 7.50)	7.00 (3.58, 8.00)	Z=-0.680	0.497
Carbohydrate Antigen 199 (ng/mL)	37.90 (25.60, 43.20)	35.60 (32.38, 44.10)	Z=-0.551	0.581
Alanine Aminotransferase (U/L)	61.00 (26.00, 67.00)	40.50 (33.00, 65.25)	Z=-0.708	0.479
Aspartate Aminotransferase (U/L)	50.00 (20.00, 77.00)	41.00 (20.75, 74.50)	Z=-0.094	0.925
Gamma-glutamyl Transferase (U/L)	75.00 (44.00, 112.00)	97.50 (46.75, 110.25)	Z=-0.266	0.790
Alkaline Phosphatase (U/L)	90.00 (65.00, 146.00)	73.50 (66.00, 141.75)	Z=-0.684	0.494

### 3.3 Spectral CT quantitative parameters

A comparison of the preoperative spectral CT quantitative parameters is presented in **Table 3**. In the arterial phase, the NIC, λ-HU, and Z-eff values were significantly higher in the Incomplete Ablation group compared to the Complete Ablation group (all P<0.001). In the portal venous phase, there were no statistically significant differences in NIC, λ-HU, or Z-eff between the two groups (all P>0.05).

**Table 3:** Comparison of spectral quantitative parameters.

Variables		Complete ablation group (n=81)	Incomplete ablation group (n=28)	Statistic	*P*
NIC	Arterial Phase	0.17 ± 0.05	0.23 ± 0.07	t=-4.847	<0.001
	Venous Phase	0.45 ± 0.09	0.47 ± 0.10	t=-1.499	0.137
λ-HU	Arterial Phase	2.22 ± 0.64	3.40 ± 1.01	t=-5.821	<0.001
	Venous Phase	2.15 ± 0.48	2.33 ± 0.63	t=-1.642	0.104
Z-eff	Arterial Phase	8.62 ± 0.52	9.13 ± 0.61	t=-4.311	<0.001
	Venous Phase	8.35 ± 0.45	8.43 ± 0.49	t=-0.698	0.487

NIC, Normalized Iodine Concentration; λ-HU, Lambda derived from Hounsfield Units; Z-eff, Effective Atomic Number.

### 3.4 Univariate and multivariate logistic regression analysis

To further investigate the risk factors for incomplete ablation, univariate logistic regression analysis was first performed on variables that showed statistical significance in the univariate analysis. The results are shown in **Table 4**. All these variables were significant risk factors for incomplete ablation (all P<0.01). Subsequently, these variables were entered into a multivariate logistic regression model for stepwise analysis. The results are presented in **Table 5**. All six variables were identified as independent predictors of incomplete ablation (all P<0.05). Among them, adjacency to major intrahepatic vessels had the highest odds ratio (OR = 12.898, 95% CI: 1.339-124.214), indicating its most prominent predictive value for the risk of incomplete ablation.

**Table 4:** Univariate logistic regression analysis of incomplete ablation-related factors.

Variables	β	S.E	Z	*P*	OR (95%CI)
Tumor size (>3 cm)	1.853	0.482	3.846	<0.001	6.381 (2.482-16.407)
Adjacent to major intrahepatic vessels	1.258	0.458	2.746	0.006	3.519 (1.433-8.640)
AFP (>100 ng/mL)	1.240	0.475	2.610	0.009	3.456 (1.362-8.769)
NIC (Arterial Phase)	1.878	0.474	3.958	<0.001	6.538 (2.580-16.566)
λ-HU (Arterial Phase)	2.012	0.441	4.566	<0.001	7.479 (3.153-17.738)
Z-eff (Arterial Phase)	1.696	0.460	3.684	<0.001	5.450 (2.211-13.432)

**Table 5:** Multivariate logistic regression analysis of incomplete ablation-related factors.

Variables	β	S.E	Z	*P*	OR (95%CI)
Tumor size (>3 cm)	2.140	0.929	2.304	0.021	8.502 (1.377-52.503)
Adjacent to major intrahepatic vessels	2.557	1.156	2.213	0.027	12.898 (1.339-124.214)
AFP (>100 ng/mL)	2.271	1.010	2.249	0.025	9.691 (1.339-70.146)
NIC (Arterial Phase)	1.734	0.682	2.541	0.011	5.662 (1.486-21.568)
λ-HU (Arterial Phase)	1.464	0.540	2.709	0.007	4.324 (1.499-12.470)
Z-eff (Arterial Phase)	1.633	0.749	2.181	0.029	5.121 (1.180-22.224)

### 3.5 Construction and performance evaluation of the incomplete ablation prediction model

**Figure 2:**
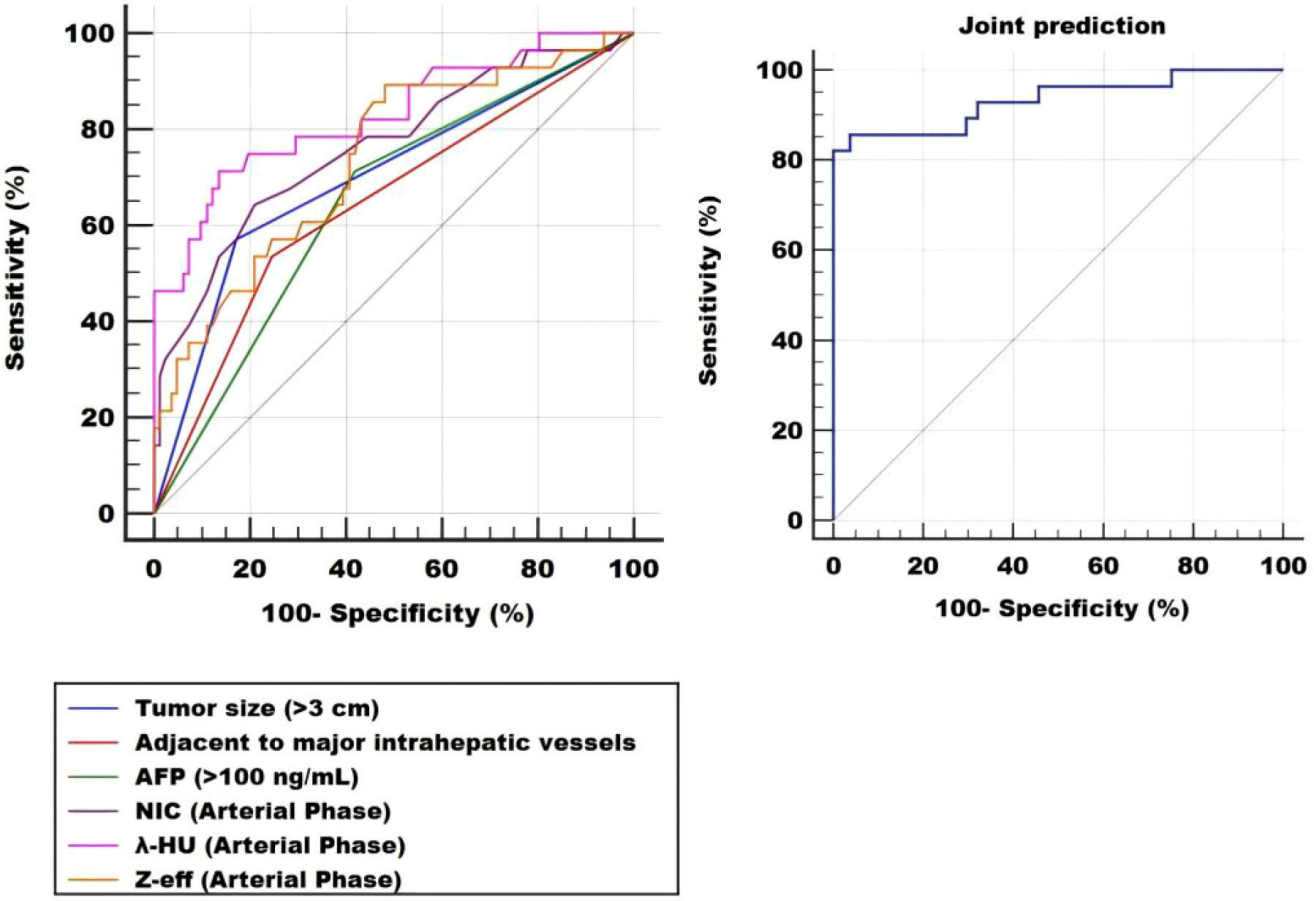
ROC curves of different indicators for predicting incomplete ablation.

**Figure 3:**
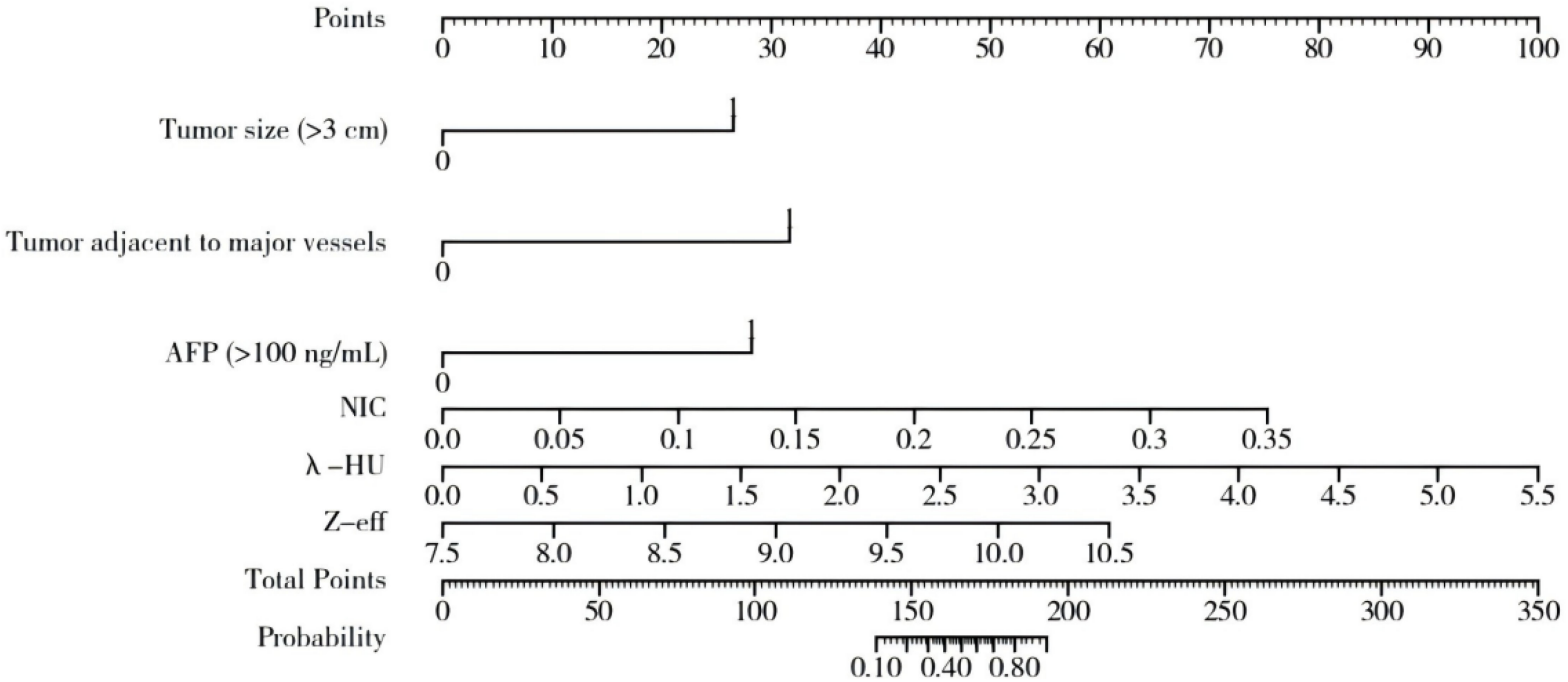
Nomogram for predicting incomplete ablation after radiofrequency ablation of liver tumors.

A combined prediction model was constructed based on the six independent predictive factors identified through the multivariate logistic regression analysis. The predictive performance of each individual indicator and the combined model was evaluated by plotting ROC curves. The results are presented in **Table 6** and **Figure 2**. Among the individual predictive indicators, the arterial phase λ-HU yielded the highest AUC of 0.829 (95% CI: 0.745-0.894), followed by arterial phase NIC (AUC = 0.763, 95% CI: 0.672-0.839) and arterial phase Z-eff (AUC = 0.735, 95% CI: 0.642-0.815). Among the clinical factors, tumor size achieved an AUC of 0.699 (95% CI: 0.604-0.783). The combined prediction model, incorporating all six factors, demonstrated excellent predictive capability, with an apparent AUC reaching 0.933 (95% CI: 0.869-0.972). This performance was significantly superior to that of any single indicator (P<0.001). Following internal validation using 10-fold cross-validation (repeated 5 times), the cross-validated AUC was 0.924 (95% CI: 0.886-0.942), confirming the model’s generalizability while indicating that the original apparent AUC was slightly optimistic. A nomogram was constructed to facilitate clinical use (**Figure 3**).

**Table 6:** The AUC of different indicators for predicting incomplete ablation.

Variables	AUC	SE^a^	95%CI^b^	*Z*	*P*
Tumor size (>3 cm)	0.699	0.052	0.604-0.783	3.825	<0.001
Adjacent to major intrahepatic vessels	0.644	0.054	0.547-0.734	2.689	0.007
AFP (>100 ng/mL)	0.647	0.052	0.550-0.736	2.860	0.004
NIC (Arterial Phase)	0.763	0.057	0.672-0.839	4.653	<0.001
λ-HU (Arterial Phase)	0.829	0.050	0.745-0.894	6.606	<0.001
Z-eff (Arterial Phase)	0.735	0.056	0.642-0.815	4.187	<0.001
Joint prediction	0.933	0.034	0.869-0.972	12.733	<0.001

^a^ DeLong etal., 1988.

^b^ Exact Binomial Test.

## 4 Discussion

This study systematically evaluated the correlation between preoperative spectral CT quantitative parameters, clinical indicators, and the status of complete ablation following RFA. The results demonstrate that a maximum tumor diameter>3 cm, preoperative AFP level>100 ng/mL, tumor adjacency to major intrahepatic vessels, and elevated arterial phase NIC, λ-HU, and Z-eff are independent risk factors for predicting incomplete ablation. A combined predictive model constructed based on these factors exhibited excellent discriminative performance, achieving an AUC as high as 0.933, which was significantly superior to any single indicator. This provides robust evidence-based support for the precise pre-treatment prediction of ablation outcomes and the optimization of surgical pathway planning in clinical practice.

This study confirmed that a maximum tumor diameter>3 cm is a strong independent predictor of incomplete ablation. Tumors with a diameter ≤ 3 cm have the highest rates of complete ablation and durable local control (19). When tumor size exceeds 3–4 cm, the likelihood of incomplete treatment and recurrence increases, often necessitating combined therapeutic strategies (20). For tumors larger than 5 cm in diameter, ablation alone is generally insufficient, and other approaches such as embolization, surgery, or systemic therapy are preferred (21). Tumor adjacency to major intrahepatic vessels has been identified as the most significant risk factor, with a multivariate OR of 12.898, highlighting its prominent role in predicting ablation failure. This is primarily attributable to the blood flow-related “heat-sink effect” (22). The liver has a rich blood supply. Large-caliber blood vessels adjacent to a tumor act as a continuously operating cooling system; the flowing blood rapidly dissipates the heat generated by the radiofrequency electrode, preventing effective temperature elevation in the tumor tissue near the vessel and creating “heat-sink cold spots” where residual tumor is likely (23). Potential confounding factors, including tumor type, operator experience, and procedural details, warrant careful consideration. In this study, the distribution of pathological types (hepatocellular carcinoma, intrahepatic cholangiocarcinoma, and liver metastases) was comparable between the complete and incomplete ablation groups, and all patients underwent RFA using a standardized protocol under ultrasound guidance by experienced interventional radiologists (each with >5 years of experience). Therefore, the impact of these factors on the primary outcome is likely limited. Nevertheless, operator-dependent variations in needle placement, ablation time, and power settings cannot be fully excluded in a retrospective design, and these factors may influence the achievement of an adequate ablation margin, particularly for tumors in challenging locations.

Preoperative serum AFP level>100 ng/mL, a biological marker, was also confirmed as an independent predictive factor. Studies by Luo et al. (24) and Zhuang et al. (25) have similarly demonstrated that a high AFP level is an independent risk factor for tumor recurrence after RFA, further supporting our conclusion. Tumors with elevated AFP levels often exhibit more active metabolism and richer blood supply. This not only increases the risk of the heat-sink effect but may also indicate that the tumor cells possess greater tolerance or repair capacity against thermal injury. Furthermore, the microenvironment of such tumors may be more invasive, with tumor cells more prone to infiltrating surrounding tissues. This makes it difficult to define clear ablation margins, and microscopic foci of infiltration may persist even when the ablative zone covers the visible lesion on imaging. Therefore, patients with significantly elevated preoperative AFP should be regarded as a high-risk group for ablation therapy. This necessitates more detailed preoperative imaging assessment and more intensive, long-term postoperative follow-up to enable early detection of potential residual or recurrent disease.

Our findings reveal that during the arterial phase, the NIC, λ-HU, and Z-eff values were significantly higher in the incomplete ablation group compared to the complete ablation group, and these emerged as independent risk factors for predicting incomplete ablation. NIC is a direct indicator reflecting tissue blood supply and perfusion levels, with its value being proportional to the iodine concentration per unit volume within the tissue (26). Malignant liver tumors, particularly hepatocellular carcinoma, typically exhibit hypervascular characteristics, demonstrating pronounced enhancement during the arterial phase. A higher NIC indicates abnormally rich and disorganized arterial blood supply within the tumor. This highly vascularized state may, on one hand, impede adequate local temperature elevation by enhancing the heat-sink effect. On the other hand, it is often associated with rapid tumor growth and invasive behavior (27). Research by Luo et al. (28) suggests that arterial phase NIC from spectral CT can reflect tumor-derived angiogenesis in hepatocellular carcinoma and serves as a potential biomarker for predicting early recurrence. λ-HU reflects the rate of change in a material’s CT value with varying X-ray energy levels, primarily determined by the material’s atomic number. In contrast-enhanced scans, iodine is the main high-atomic-number element influencing tissue attenuation. Since the photoelectric effect is more pronounced at lower energy levels, tissues rich in iodine exhibit a steeper spectral curve, corresponding to a higher λ-HU value (29). Therefore, λ-HU essentially reflects the tumor’s iodine uptake capacity from another dimension, sharing intrinsic consistency with NIC and both pointing to the tumor’s blood supply status. Z-eff, a parameter representing the average atomic number of elements in a composite material, shows increased values in the arterial phase of enhanced scans, which is also directly attributed to iodine accumulation within the tumor tissue (30). Consequently, elevated arterial phase Z-eff also serves as an independent risk factor predicting incomplete ablation. The high consistency in the predictive direction among these three spectral quantitative parameters, derived from different physical principles and calculation methods, demonstrates that the tumor’s hyper-perfusion state during the arterial phase is a crucial functional imaging characteristic leading to RFA treatment failure. Research by Li et al. (31) showed that all spectral quantitative parameters (λ-HU, NIC, and Z-eff) in arterial phase for metastatic breast cancer axillary lymph nodes were higher than those for non-metastatic nodes, further supporting the findings of this study.

In recent years, several imaging−based prediction models have been developed to anticipate incomplete ablation or local tumor progression following RFA for liver tumors. For instance, deep learning radiomics based on contrast−enhanced CT has shown excellent performance (AUC 0.906) in predicting proliferative HCC and stratifying RFA patients into high− and low−risk groups (32). Similarly, delta−radiomics analysis using multi-phase MRI achieved an AUC of 0.893 for predicting early recurrence after percutaneous thermal ablation (33). Spectral CT, with its ability to provide material decomposition and quantitative parameters such as NIC, λ−HU, and Z−eff, offers advantages over conventional imaging by reflecting tumor hemodynamics and biological behavior more directly. Recent studies have further demonstrated that combining serum biomarkers (AFP, AFP−L3) with radiomics features yields superior predictive performance (AUC up to 0.897) compared to either modality alone (34). However, most existing studies have focused on single−modality parameters or conventional clinical factors, and few have integrated spectral CT quantitative parameters with clinical risk factors into a combined predictive model for RFA completeness. Our study not only confirms the independent predictive value of arterial−phase NIC, λ−HU, and Z−eff but also constructs a multi−parameter model that significantly outperforms any single indicator, filling an important gap in the literature regarding preoperative prediction of RFA outcomes. Although primary liver cancer and liver metastases may theoretically require different ablation margins due to distinct biological behaviors, the distribution of these two tumor types was comparable between the complete and incomplete ablation groups, and all patients received the same ablation protocol with a target margin of ≥1 cm. Nevertheless, the lack of margin stratification by tumor origin represents a limitation of this study and should be addressed in future prospective designs.

## 5 Study limitations

First, this study is a single-center, retrospective analysis, which inevitably introduces selection bias. The generalizability and external validity of the findings may be somewhat limited and require validation in multi-center settings across different regions. Second, although the total sample size met statistical requirements, the number of cases in the incomplete ablation group was relatively small (n=28), resulting in an events-per-variable ratio below the recommended threshold and increasing the risk of model overfitting. Although internal validation using cross-validation and bootstrap showed acceptable performance, external validation is essential. Third, the efficacy evaluation in this study used contrast-enhanced MRI at one month postoperatively as the gold standard, which primarily reflects immediate ablation efficacy and does not track long-term outcomes. Fourth, formal calibration metrics (e.g., Hosmer-Lemeshow test, calibration plots) were not performed due to the limited number of incomplete ablation events (n=28), as such analyses would be underpowered and potentially misleading in small-sample settings. Calibration assessment should be addressed in future large-scale validation studies. Fifth, the predictive model requires external validation in independent cohorts before widespread clinical adoption.

## 6 Conclusion

Through this retrospective analysis, we found that tumor size, location, preoperative AFP level, and spectral CT quantitative parameters are key indicators for predicting the completeness of RFA in liver tumors. The combined predictive model integrating these multi-dimensional indicators demonstrates high predictive value. It assists in identifying patients at high risk for incomplete ablation prior to treatment, thereby providing an objective basis for formulating individualized therapeutic strategies and optimizing ablation protocols.

## Data Availability

The original contributions presented in the study are included in the article/supplementary material. Further inquiries can be directed to the corresponding author.
